# Decoder-seq: a technology for high sensitivity, high resolution, and low-cost spatial RNA sequencing

**DOI:** 10.52601/bpr.2024.240903

**Published:** 2024-06-30

**Authors:** Siquan Li, Jin Li, He Huang

**Affiliations:** 1 Department of Urology, The First Affiliated Hospital of Nanchang University, Nanchang 330006, China; 2 Institute of Metabolism and Integrative Biology, State Key Laboratory of Genetic Engineering, School of Life Sciences and Zhongshan Hospital, Fudan University, Shanghai 200438, China

The spatially resolved transcriptomics (SRT) technology serves as a powerful tool for delineating the spatial gene expression patterns of tissues, revealing cellular composition, distribution and interactions. Its applications span across various research fields such as embryonic development, neuroscience, and diseases, attracting widespread attention. Existing SRT technologies mainly include: (1) imaging-based *in situ* hybridization or sequencing (Chen *et al*. 2015; Zeng *et al*. 2023); and (2) spatial barcoded array-based sequencing technology (Stahl *et al*. 2016; Vickovic *et al*. 2019). Compared to the former, the latter can capture mRNAs at a tissue scale and identify their type, abundance, and spatial locations through next-generation sequencing (NGS), which provides an unbiased, straightforward, and high-throughput approach for SRT analysis. The key step in spatial barcoded array-based sequencing technology involves using the array to encode tissue mRNAs *in situ*, directly determining the resolution and sensitivity of such methods. To reach a cellular or subcellular spatial resolution, researchers have fabricated barcoded DNA arrays by introducing randomly generated DNA micro/nanoballs or clusters. However, this barcoding strategy requires time-consuming and expensive decoding processes to determine the random spatial location of balls or clusters. The sensitivity of gene detection is another crucial issue that needs to be concerned with spatial barcoded array-based sequencing technology. A low sensitivity may introduce gene detection bias and miss important low-abundance transcripts, as well as require deeper sequencing depth to ensure sufficient gene coverage for SRT analysis. This restricts the application scope of the method and increases sequencing costs to some extent. Therefore, the current spatial barcoded array-based sequencing technology faces challenges in balancing gene detection sensitivity and spatial resolution. The complex process of preparing barcoded DNA arrays and their high cost also pose certain technical barriers for many researchers and communities.

A recent study by Cao *et al*. established an assay called reported Decoder-seq (Cao *et al*. 2024), which is a flexible and accessible spatial barcoded array-based sequencing technology that enables high sensitivity, high resolution and low-cost SRT analysis (Fig. 1). By assembling spherical dendrimers on glass slides, 3D nanostructured substrates with abundant amino functional groups were formed. This significantly enhances the modification density of spatial DNA barcodes by about an order of magnitude compared to other substrates, while also increasing the distance between barcodes and substrates, thereby improving accessibility for effective mRNA capture. To streamline the fabrication of a barcoded DNA array, a microfluidics-based barcoding strategy was employed to generate deterministic and combinatorial *X*_*i*_*Y*_*j*_ coordinates on 3D dendrimeric substrates. A pair of microfluidics chips with channels perpendicular to each other were designed and sequentially placed on the slide to introduce *X* and *Y* sets of barcode solutions. By adjusting the number and width of the microchannel, the barcoded DNA arrays with different capture areas and spatial resolutions (50, 25, 15 and 10 μm) were flexibly generated. Moreover, this deterministic combinatorial barcoding strategy significantly reduces the variety of DNA barcodes without requiring decoding steps and greatly reduces costs. Compared to other similar technologies, Decoder-seq is affordable and accessible, making it easily adoptable by other labs using readily available reagents and instruments.

**Figure 1:**
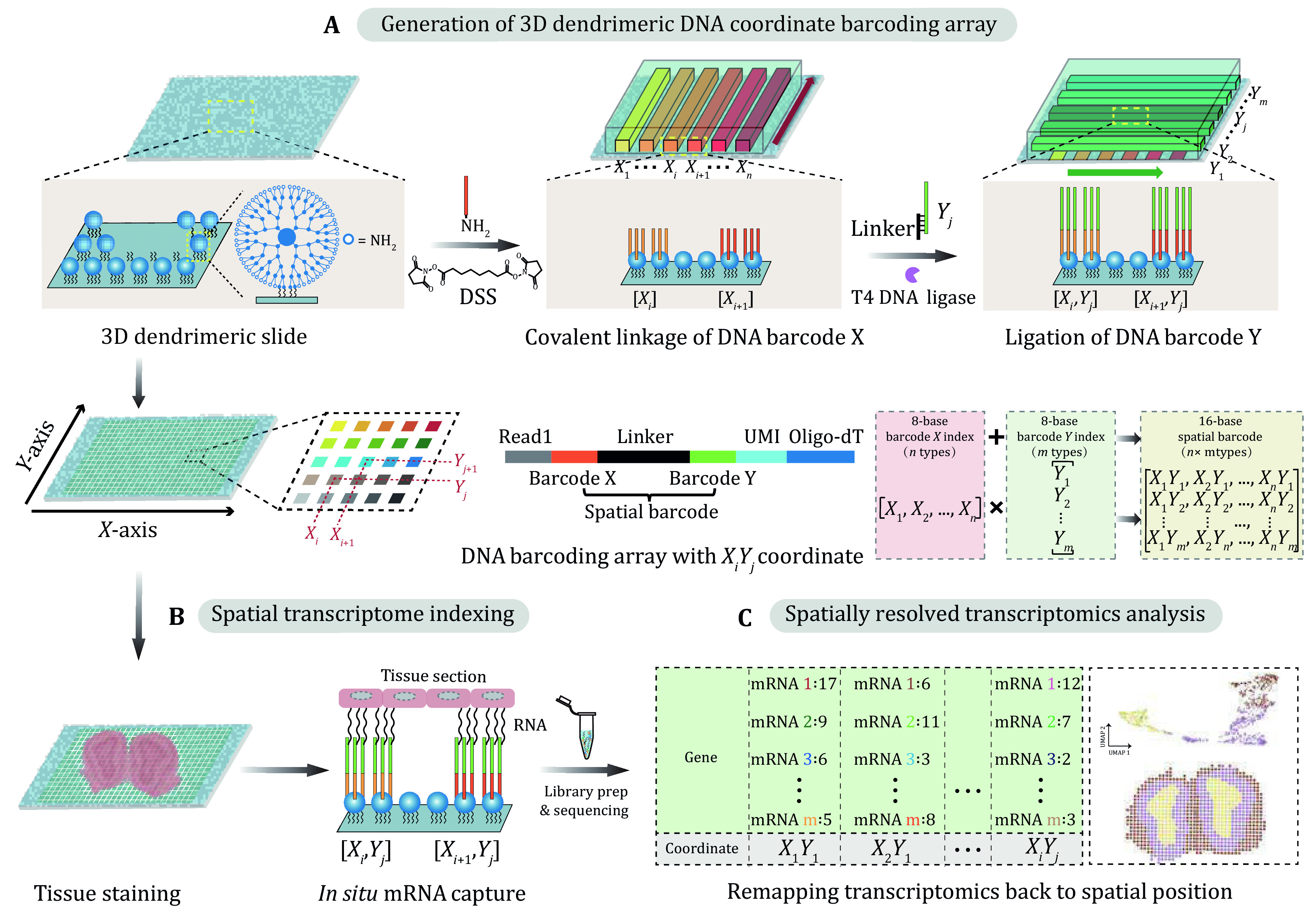
Workflow of Decoder-seq referring from Cao *et al*. (2024). **A** Generation of a 3D spatial barcoded DNA array by combining 3D dendrimeric substrate with microfluidics-based combinatorial barcoding strategy. **B** Spatial transcriptome indexing. C SRT analysis through bioinformatic

Using model tissues of mouse olfactory bulbs (MOBs), Cao *et al*. have successfully demonstrated the high gene detection sensitivity of Decoder-seq. The detection sensitivity of Decoder-seq was approximately (68.9 ± 15.6)% compared to *in situ* sequencing technology. Decoder-seq with near cellular spatial resolution (15 μm) detected 40.1 UMI and 14.7 genes per μm^2^, surpassing other cutting-edge spatial barcoded array-based sequencing technologies. Compared to commercial 10× Visium, Decoder-seq identified a five-fold increase in lowly-expressed olfactory receptor (*Olfr*) genes. More importantly, the 15 μm-spot Decoder-seq observed a unique layer enriched spatial patterns of two *Olfr* genes that were not discernible using other similar methods. This indicates that the high gene detection sensitivity is advantageous for spatial localizing of lowly-expressed genes, such as *Olfr*, enabling analysis of previously challenging basic physiological activities like olfactory mechanism in a high-throughput manner. Besides, based on an image-based cell segmentation algorithm and cell-type deconvolution, Decoder-seq successfully generated a spatial single-cell atlas of the mouse hippocampus that faithfully aligned with the ground truth of the HE image. Finally, Decoder-seq investigated the spatial heterogeneity and potential tumor invasion behaviors of two subtypes of human renal cell carcinoma (RCC), predicting clinical outcomes of RCC patients by identifying a panel of gradient-expressed epithelial-mesenchymal transition-related genes. This highlights the potential of Decoder-seq for analyzing complex tissue samples.

In sum, the establishment of the Decoder-seq platform offers unique opportunities for researchers to understand the basic biological principles as well as deep pathological mechanisms. The fabrication process of barcoded DNA arrays is characterized by its simplicity and cost-effectiveness, making it replicable and affordable for adoption of the Decoder-seq technology in other laboratories. In contrast to other high-resolution SRT technologies, Decoder-seq effortlessly achieves single-cell resolution without imposing high technical barriers. The innovative design of Decoder-seq incorporates 3D nanostructured dendrimeric substrates and microfluidics-based barcoding strategy, enhancing RNA capture efficiency to surpass similar technologies in the performance of gene detection. The design also has remarkable versatility in its compatibility with other spatial omics or multi-omics technologies. It is worth mentioning that the high density of modifications in Decoder-seq has the potential to address the current issue of sparse data in omics or multi-omics technologies, where challenges arise from low molecular capture efficiency or competition for capture from multiple molecules.

## Conflict of interest

Siquan Li, Jin Li and He Huang declare that they have no conflict of interest.
